# Higher plasma drug levels in elderly people living with HIV treated with darunavir

**DOI:** 10.1371/journal.pone.0246171

**Published:** 2021-02-04

**Authors:** Erika Tyrberg, Arvid Edén, Jaran Eriksen, Staffan Nilsson, Carl Johan Treutiger, Anders Thalme, Åsa Mellgren, Magnus Gisslén, Lars-Magnus Andersson

**Affiliations:** 1 Department of Infectious Diseases, Institute of Biomedicine, Sahlgrenska Academy, University of Gothenburg, Gothenburg, Sweden; 2 Region Västra Götaland, Department of Infectious Diseases, Sahlgrenska University Hospital, Gothenburg, Sweden; 3 Division of Clinical Pharmacology, Department of Laboratory Medicine, Karolinska Institute, Stockholm, Sweden; 4 Department of Infectious Diseases/Venhälsan, Stockholm South General Hospital, Stockholm, Sweden; 5 Mathematical Sciences, Chalmers University of Technology, Gothenburg, Sweden; 6 Department of Infectious Diseases, Karolinska University Hospital, Stockholm, Sweden; 7 Clinic of Infectious Diseases, South Älvsborg Hospital, Borås, Sweden; University of Tennessee Health Science Center College of Pharmacy Memphis, UNITED STATES

## Abstract

**Background:**

The proportion of elderly people living with HIV-1 (PLHIV) is rising. In older patients, comorbidities and concomitant medications are more frequent, increasing the risk of potential drug-drug interactions (PDDIs). Data on the pharmacokinetics of ART in individuals aged ≥ 65 years of age are scarce. We compared plasma drug levels of ART, PDDIs, and side-effects in PLHIV aged ≥ 65 years of age, with controls ≤ 49 years of age.

**Methods:**

Patients ≥ 65 years of age and controls ≤ 49 years of age, all of whom were on stable treatment with atazanavir (ATV), darunavir (DRV), or efavirenz (EFV) were included cross-sectionally. Plasma drug levels of ART were analyzed, comorbidities, concomitant medication, adherence, and side-effects recorded, and PDDIs analyzed using drug interactions databases.

**Results:**

Between 2013 and 2015, we included 100 individuals ≥ 65 years of age (study group) and 99 controls (≤ 49 years of age). Steady-state DRV concentrations were significantly higher in the study group than in the control group (*p* = 0.047). In the ATV group there was a trend towards a significant difference (*p* = 0.056). No significant differences were found in the EFV arm. The DRV arm had a higher frequency of reported side-effects than the ATV and EFV arms in the study group (36.7% vs. 0% and 23.8% respectively (*p* = 0.014), with significant differences between DRV vs. ATV, and EFV vs. ATV).

**Conclusions:**

Higher steady-state plasma levels of DRV and ATV (but not EFV) were found in PLHIV aged ≥ 65 years of age, compared to controls ≤ 49 years of age.

## Introduction

Antiretroviral therapy (ART) has dramatically changed the life expectancy of people living with HIV (PLHIV). HIV can now be considered a chronic infection, and the expected life span of PLHIV who receive efficient treatment is comparable to HIV-negative individuals [1, 2]. As a consequence, an increasing number of PLHIV are of older age. For example, in 2018 51% of PLHIV in the US were 50 years of age or older [3].

The risk of developing age-related and lifestyle-related diseases increases with age. PLHIV are, in addition, at higher risk of non-infectious comorbidities compared to the general population [4–6]. Furthermore, elderly people are, in general, at higher risk of adverse events to medications and may require lower doses of medications than recommended for younger individuals [7, 8]. It has been reported that PLHIV above 50 years of age have more concomitant medications and a higher risk of potential drug-drug interactions (PDDIs) compared to PLHIV below 50 years of age [9, 10].

Since the mid 1990s the standard regime for HIV treatment is two nucleoside reverse-transcriptase inhibitors (NRTIs) combined with a third agent from another drug class (most commonly a nucleoside reverse-transcriptase inhibitor (NNRTI), a protease inhibitor (PI) or an integrase inhibitor (INSTI). A dolutegravir (INSTI) or Efavirenz (NNRTI) containing regimen is recommended by WHO as first line treatment today [11]. In the Swedish setting a dolutegravir or darunavir containing regimen is recommended by the Swedish Reference Group for Antiviral Therapy [12]. Neither WHO nor Sweden have specific treatment recommendations for elderly PLHIV.

Scientific data on the pharmacokinetics of PIs and NNRTIs in individuals 65 years of age and older are scarce. The primary objective of this study was to investigate differences in steady-state plasma drug levels of ATV, DRV and EFV in PLHIV ≥ 65 years of age as compared to PLHIV ≤ 49 years of age. Secondary objectives were to study differences in self-reported side-effects, concomitant chronic diseases and medications, and PDDIs.

## Methods

PLHIV who were followed at four HIV centers in Sweden: the Department of Infectious Diseases at Sahlgrenska University Hospital in Gothenburg; the Department of Infectious Diseases at South Älvsborg Hospital in Borås; the Department of Infectious Diseases at Karolinska University Hospital Huddinge in Stockholm; and the Department of Infectious Diseases at Stockholm South General Hospital in Stockholm, and met the inclusion criteria (age, 65 years of age or older for the study group or 49 years of age or younger for the control group; and on stable ART containing atazanavir (ATV), darunavir (DRV) or efavirenz (EFV) for more than 6 months) were eligible for inclusion in this cross-sectional study. On the day of inclusion, a blood sample for analysis of plasma drug level was taken, and concomitant medications (including non-prescription drugs and herbal supplements) and any side-effects related to ART were recorded in a standardized questionnaire, S1 File. Blood samples drawn between 6 to 36 hours after last dose of medication was included in the analysis of steady-state drug levels and adjusted for time with ANCOVA analysis. Adherence was recorded using a modified ACTG adherence questionnaire [13]. Any missed dose during the preceding 4 days was considered as non-adherence.

Comorbidities were registered by structured medical record reviews. PDDIs were analyzed using the Liverpool University HIV drug interactions [14] and Janusmed [15] webtools. The Liverpool University HIV drug interactions database definitions for PDDIs were used and red flag (drugs should not be co-administered) and orange flag (a potential interaction that may require dose monitoring, alteration of drug dosage or timing of administration) interactions were included in the analysis. If there was an interaction between a comedication and both the PI and the booster, the interaction was counted as one interaction in the analysis. Individuals taking DRV b.i.d. or ATV without ritonavir booster were excluded from the analysis of plasma drug levels.

All study participants gave their written informed consent and ethics approval for the study was granted by the Research Ethics Committee at Gothenburg University.

### Laboratory analyses

Plasma samples were frozen at –70°C immediately after sampling until analysis. Drug levels were analyzed using a reverse-phase High Pressure Liquid Chromatography (HPLC) with ultraviolet (UV) detection at the routine pharmacology analytical laboratory at Karolinska University Hospital, Huddinge in Stockholm, Sweden. The method was CAP (College of American Pathologists) and Swedac accredited and has been described elsewhere [16]. Routine clinical methods were used to analyze CD4 cell count, liver enzymes and creatinine according to local laboratory standards.

### Statistical analyses

Differences in plasma drug levels were analyzed with ANCOVA (adjusting for time) with log-transformed concentrations of ATV, DRV, and EFV. Chi-square test and Fisher’s exact test were used to compare the frequencies of side-effects, CD4/CD8 ratios, and AIDS diagnosis as appropriate. Mann Whitney U-test and Kruskal Wallis test (with Bonferroni correction for multiple tests) were used to compare frequencies of concomitant medications and PDDIs. A *p*-value < 0.05 was considered statistically significant. All statistical analyses were performed using SPSS version 25 (IBM SPSS Statistics, Armonk, NY, USA) or Prism version 8.0 (Graphpad Software Inc., La Jolla, CA, USA).

## Results

One hundred and seventy-two individuals 65 years of age or older were eligible for inclusion and were asked to participate in the study at the four sites. Between November 2013 and August 2015, 100 individuals were enrolled in the study group (ATV n = 19; DRV n = 35; EFV n = 46) and 99 individuals in the control group (ATV n = 18; DRV n = 37; EFV n = 44). Baseline characteristics are listed in Table 1. Three individuals had HIV RNA blips (HIV RNA 59–156 copies/mL) at inclusion; all other patients had HIV RNA levels < 50 copies/mL at inclusion. Twenty-seven patients were excluded from the plasma drug level analysis: 15 individuals received DRV b.i.d. and 9 were treated with ATV, either unboosted or with dosing not according to clinical standards. Three were excluded from the plasma drug level analysis due to sample management (elapsed time since last dose less than 6 hours, or elapsed time since last dose unknown). Patients included in the plasma drug level analysis received DRV/r 800/100 mg, ATV/r 300/100 mg, or EFV 600 mg q.d. There was a significant difference in ALT levels between study and control group in the ATV arm, however the majority of subjects had ALT within the normal range. The study group had a lower glomerular filtration rate (GFR) in all arms compared to controls, although within the normal range.

**Table 1:** Baseline characteristics.

	ATV			DRV			EFV		
	Study n = 19	Control n = 18	*p*	Study n = 35	Control n = 37	*p*	Study n = 46	Control n = 44	*p*
Age (median [IQR])	68 (66–70)	46 (40.75–47.5)		68 (67–72)	45 (37.5–47)		69 (67–72)	43 (37–46)	
Gender (M/F) (n)	13/6	17/1	0.09	33/2	32/5	0.43	41/5	38/6	0.76
BMI (median [IQR])	24.3 (22.3–27.2)	25.3 (23.9–27.9)	0.28	25.7 (23.0–26.9)	23.5 (22.5–26.0)	0.17	24.4 (21.9–27.8)	24.2 (22.1–28.5)	0.65
GFR (ml/min) (median [IQR])	83.7 (71.0–96.8)	118.0 (109.9–134.4)	<0.001	86.1 (55.4–95.3)	110.4 (97.0–135.8)	<0.001	79.9 (67.6–98.5)	124.3 (116.0–145.2)	<0.001
ALT (μkat/L) (median [IQR])	0.40 (0.30–0.55)	0.63 (0.52–0.75)	0.017	0.32 (0.24–0.48)	0.41 (0.31–0.55)	0.14	0.48 (0.33–0.56)	0.55 (0.38–0.85)	0.056
CD4 cell count (median [IQR])	650 (370–730)	730 (490–912.5)	0.13	560 (420–650)	600 (465–830)	0.54	535 (380–687.5)	575 (407.5–750)	0.39
CD4 cell count Nadir (median [IQR])	130 (69–223)	241 (187–337)	0.001	185 (90–281)	218 (40–310)	0.97	208 (158–263)	219 (167–303)	0.49
**Backbone 3TC/ABC**	9	14	0.09	11	14	0.63	23	12	0.03
**Backbone FTC/TDF**	10	4	0.09	5	12	0.03	23	30	0.09
Backbone other**	0	0	NA	19	9	0.02	0	2	0.24
Comorbidities (n) (median [IQR])	2 (1–4)	2 (0.25–3)	0.31	3 (2–5)	1 (1–3)	0.001	3 (2–4.25)	1 (0–2)	<0.001

** Other backbones: RAL, ETV, RAL + maraviroc, RAL + EFV, RAL + DRV, LPV/r, RAL + 3TC, 3TC, DTG + 3TC, RPV, no backbone.

**Fig 1:**
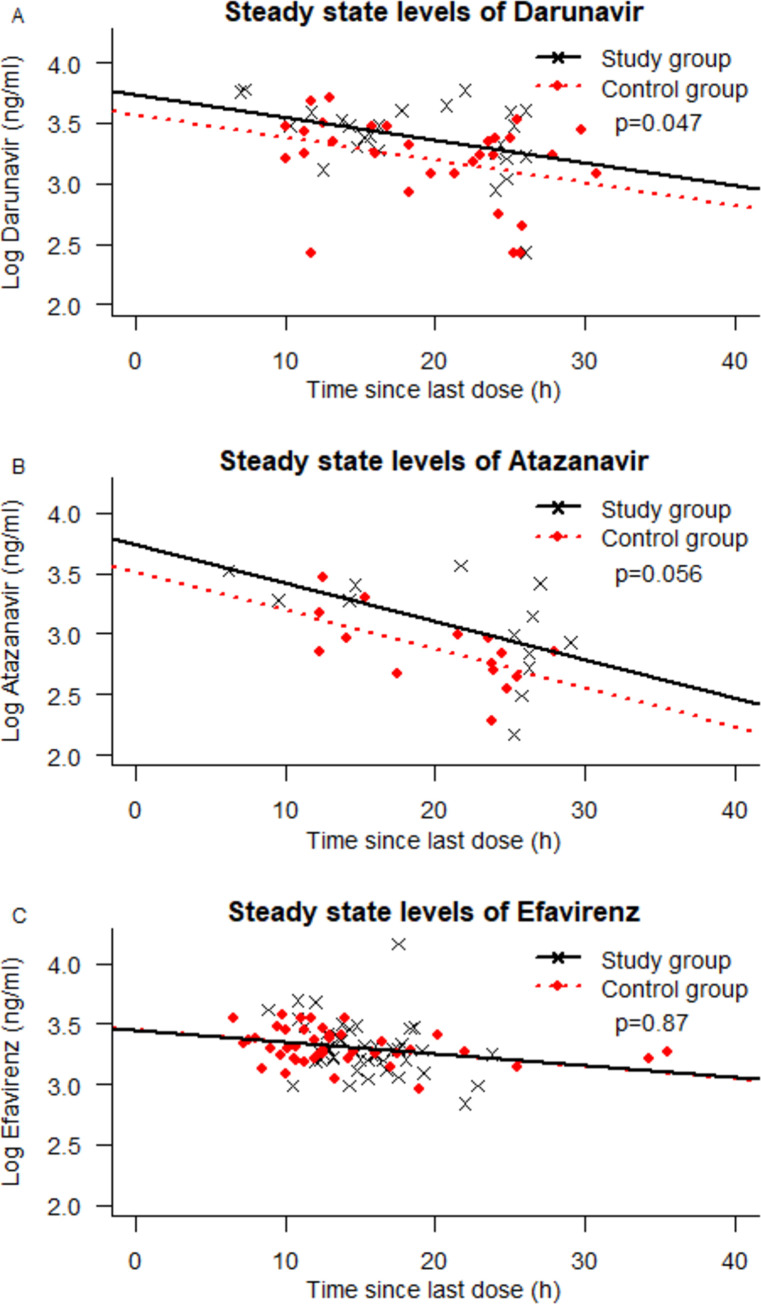
Steady-state levels of ART. Steady-state levels in plasma of A) Darunavir (*p* = 0.047), B) Atazanavir (*p* = 0.056), and C) Efavirenz (*p* = 0.87) in individuals 65 years of age or older (study group) and individuals 49 years of age or younger (control group).

The steady-state DRV concentrations were significantly higher in the study group (n = 25) compared to the control group (n = 30) (*p* = 0.047), Fig 1. The geometric mean was 48% higher in the study group than in the control group. The analysis of the ATV arm (study group n = 19, control group n = 18) showed a difference in steady state-levels (geometric mean 69% higher in the study group), with a trend towards statistical significance (*p* = 0.056). No statistically significant difference between the groups was found in the EFV (*p* = 0.87) arm. There were no differences in self-reported adherence between the study group (96% adherent) and control group (93% adherent) (*p* = 0.537), or between different treatment arms either in the study or the control group.

**Fig 2:**
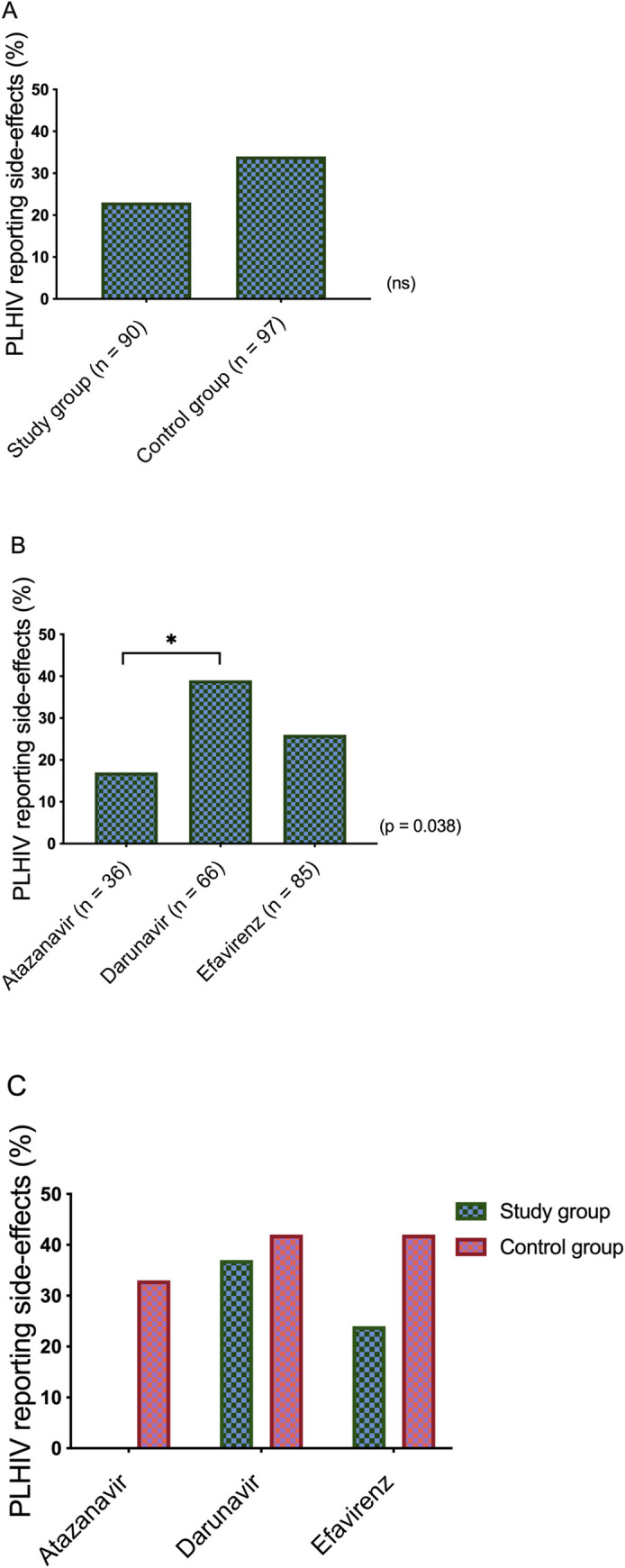
Frequency of self-reported side-effects. A: Frequency of self-reported side-effects in individuals 65 years of age or older (study group) and individuals 49 years of age or younger (control group) (ns). B: Frequency of self-reported side-effects in individuals 65 years of age or older (study group) and individuals 49 years of age or younger (control group) divided into treatment arms (*p* = 0.038). C: Frequency of self-reported side-effects divided into groups according to treatment arm and study group vs. control group.

There were no statistically significant differences in reported side-effects between the study group (23%) and the control group (34%) (*p* = 0.146), Fig 2. When dividing the groups according to drug regimen (taking the study group together with the controls), the DRV group had a higher rate of reported side-effects (ATV: 16.7%; DRV: 39.4%; EFV: 25.9%; *p* = 0.038), which was significantly different compared to the ATV arm. The difference remained when PLHIV ≥ 65 years of age were analysed separately (ATV: 0%; DRV: 36.7%; EFV: 23.8%; *p* = 0.014), with significant differences between DRV and ATV, and EFV and ATV. In the DRV arm there were no significant difference in reported side effects between the study group and the control group, (*p* = 0.80). The most commonly-reported side-effect in the DRV groups was diarrhea.

As expected, the study group had a significantly higher mean (range) number of concomitant medications, 3.7 (0–12), compared to the control group, 1.1 (0–10) (*p* < 0.001). Accordingly, the study group had significantly more PDDIs (mean (range)) 1.1 (0–6) compared to the control group, 0.3 (0–3) (*p* < 0.001). The most common PDDIs for ATV were statins and beta-blocking agents, and statins and antidepressants for DRV and EFV.

Analysis of the study group showed that the DRV arm had significantly more PDDIs (mean [range]) 1.4 (0–6) than the EFV arm 0.7 (0–3) (*p* = 0.03). The ATV arm had a mean (range) of 1.2 (0–4) PDDIs, not significantly different compared to either the DRV arm or the EFV arm (see Table 2). Notably, the use of concomitant medications was not higher in the DRV arm. Eight individuals in the study group had red flag PDDIs: DRV/r and alfuzosin (risk for severe hypotension, n = 2); DRV/r and clopidogrel (reduced effect of clopidogrel, n = 3); DRV/r and alfuzosin + clopidogrel (n = 1); ATV and budesonide (increased risk of steroid side-effects, n = 1); and ATV and lansoprazole (reduced ATV uptake, n = 1). Whereas no one in the control group had a red flag interaction.

**Table 2:** Potential drug-drug interactions.

	PDDIs (n) (mean [range])	*p*
Atazanavir	1.2 (0–4)	
Darunavir	1.4 (1–6)	0.026
Efavirenz	0.7 (0–3)	

Number of potential drug-drug interactions (PDDIs) in the study group presented by treatment regime.

There were no differences in CD4/CD8 ratios (≥ 1 or < 1) between the study group (≥ 1 n = 35 (35%)) and control group (≥ 1 n = 40 (40%)) (*p = 0*.*43*), the ATV/DRV/EFV arms, or between arms in the different groups. No differences were found in the history of AIDS defining events in the study group vs. control group. In total 38 individuals had one or more AIDS defining diagnoses, S1 Table.

## Discussion

We found a difference in the steady-state plasma drug levels of DRV in PLHIV who were ≥ 65 years of age, as compared to PLHIV < 50 years of age. To our knowledge, only one previous study has addressed the question of plasma DRV levels in elderly PLHIV [17]. In agreement with our results, the authors reported higher DRV levels in individuals > 60 years of age compared to those ≤ 40 years of age.

The difference in plasma drug levels is also consistent with earlier findings regarding other PIs, including ATV [18–20]. We noted a difference in ATV levels between elderly and younger PLHIV, with a trend towards statistical significance. The lack of significance is probably due to the small sample size. In a previous report by Avihingsanon et al., higher trough levels and higher exposure to ATV in PLHIV > 42 years of age was found compared to individuals ≤ 42 years of age, consistent with our results. This difference was more pronounced in PLHIV > 50 years of age [18]. Winston et al. also found a significant association between age and plasma drug levels of PIs [19]. We did not find any significant difference in EFV plasma drug levels in the study group compared to the control group. This is in agreement with the findings in other reports [19, 21].

There is only very limited pharmacokinetic data on ART in PLHIV older than 65 years of age. There are, however, several general age-related biological changes that may affect the metabolism of ART, e.g. decrease of liver and renal function and changes in body composition that influence the volume of distribution [22]. ATV, DRV and EFV are metabolized in the liver (ATV and DRV mainly through CYP3A4 and EFV through CYP 3A4 and CYP2B6). Only a minor portion of these drugs is eliminated through the kidneys. Therefore, the difference in drug levels in the DRV and ATV arm cannot be explained by differences in GFR. We found no clinically measurable difference in liver function measured by ALT but other changes in liver function related to age may have affected the drug levels.

Other aspects not related to metabolism may also affect the efficacy of ART. Older PLHIV have been shown to be more adherent to their treatment regimen than younger individuals [23, 24]. However, with increasing age there is a higher risk of cognitive impairment that may affect the adherence in the oldest. No difference in adherence was found in our study to support or reject either higher or lower adherence in elderly PLHIV.

Overall, we found a higher frequency of self-reported side-effects in the DRV arm, in comparison to the ATV and the EFV arms (although not significantly different from the EFV arm). The higher frequency was also present in the study group (although not statistically significant).

To the best of our knowledge this is the only study that compared the frequency of side-effects between different PIs/NNRTIs in PLHIV older than 65 years of age. A possible reason for the DRV arm having a higher frequency of side-effects might be that DRV was chosen because of extensive ART history and viral resistance, resulting in few available alternative regimes at the time of inclusion in the study and as a consequence there was a higher tolerance of side-effects. On the other hand, no difference in self-reported side effects was noted between the DRV study group and control group, even though we found a difference in steady state plasma drug levels. This observation may reflect under reporting in the study group. While it is not possible in the present study to establish a causal link, further studies are needed illuminate this issue.

PDDIs are common among PLHIV [25, 26], and the risk increases with age due to increasing frequencies of comorbidities and concomitant medications [9, 10]. Our result is in line with these earlier studies. Red flag interactions has been reported in 2% to 5.6% of PLHIV and 7.1% to 8.7% in PLHIV ≥ 65 years of age in earlier studies, similar to our findings [26–29].

ATV and DRV are both PIs and therefore they have, in general, the same PDDIs. However, they differ in regard to some frequently used drug classes e.g. beta blockers and PPIs. Since EFV is a NNRTI it has another drug interaction profile. DRV accounted for the majority of the red flag interactions found in our study, consistent with earlier findings of PLHIV in all ages [27]. Other studies have reported a higher probability of an orange or red flag PDDI in individuals treated with a PI (not restricted to those ≥ 65) [9, 28]. This is consistent with our finding that the DRV arm in the study group had a higher mean of PDDIs than the EFV arm. We did not find a significant difference between the ATV and EFV arms, however this may be due to the small sample size. The majority of the red flag interactions found were related to the concomitant use of alfuzosin, for treatment of benign prostatic hyperplasia, and/or clopidogrel, for treatment of vascular disease, which both are conditions that have a higher prevalence in older ages.

Our study has several limitations. Since we analysed steady-state plasma drug levels and not trough levels of ART, it is difficult to compare our results with trough levels in other studies. Thus, we were unable to evaluate potentially toxic plasma drug levels of ART drugs or levels below the proposed minimal effective concentrations. Patients were sampled from 6 to 36 hours after their last dose of ART. This was adjusted for in the statistical model, but there remains a risk that, because of this approach, we were not able to detect minor differences in drug levels between the study and control arms for ATV and EFV. Also, there was a difference in back-bone between PLHIV on DRV compared to ATV and EFV that may have affected self-reported side-effects. Concomitant medications may also have influenced the self-reported side-effects, though the participants were asked specifically to report side-effects related to ART. The PDDIs were calculated only for ATV, DRV or EFV regimens and therefore differences in back-bone likely did not affect the results. In addition, interactions between NRTIs and other medications are uncommon. The participants in the study were included consecutively at four sites in Sweden and it is possible that this introduced a selection bias, favoring PLHIV with frequent visits.

## Conclusion

Higher steady-state plasma levels of DRV and ATV (but not EFV) were found in PLHIV who were 65 years of age or older, as compared to controls who were 49 years of age and younger. Our findings are important for the management of elderly PLHIV and raise the question of whether regular monitoring of plasma levels and dose adjustment of DRV and other PIs is warranted in the elderly.

## Supporting information

S1 Table Aids defining diagnoses. (DOCX)

S2 Table Data set. (XLSX)

S1 File Questionnaire/case report form. (DOCX)
