# Correction: Higher plasma drug levels in elderly people living with HIV treated with darunavir

**DOI:** 10.1371/journal.pone.0251856

**Published:** 2021-05-13

**Authors:** The PLOS ONE Staff

## Notice of republication

An incorrect version of Supporting Information file S2 Table was included in the originally published article. The publisher apologizes for the error. This article was republished on April 30, 2021 to correct for this error. Please download this article again to view the correct version.
