# Supplementary material for: Higher plasma drug levels in elderly people living with HIV treated with darunavir
Source: PLoS One. 2021 Feb 4;16(2):e0246171. doi: 10.1371/journal.pone.0246171 (PMC7861408; doi:10.1371/journal.pone.0246171)
Supplement: S1 Table — (DOCX) [file pone.0246171.s001.docx]

S1 Table. Aids defining diagnoses

| **Aids defining diagnoses** | **Number of patients** |
| --- | --- |
| PCP | 15 |
| Lymphoma | 4 |
| Wasting syndrome | 2 |
| Tuberculosis | 4 |
| Kaposi’s sarcoma | 2 |
| Candida esophagitis | 1 |
| PML | 1 |
| Encephalopathy | 1 |
| Cerebral toxoplasmosis | 1 |
| PCP and candida esophagitis | 1 |
| Wasting syndrome and candida esophagitis | 1 |
| Kaposi’s sarcoma and candida esophagitis | 1 |
| Kaposi’s sarcoma and PCP | 1 |
| Candida esophagitis and CMV | 1 |
| PCP and wasting and atypical mycobacterial infection | 2 |
