# Supplementary material for: Higher plasma drug levels in elderly people living with HIV treated with darunavir
Source: PLoS One. 2021 Feb 4;16(2):e0246171. doi: 10.1371/journal.pone.0246171 (PMC7861408; doi:10.1371/journal.pone.0246171)
Supplement: S1 File — (DOCX) [file pone.0246171.s003.docx]

**Case Report Form**

Patient initials and inclusion number…………………..

Mark relevant alternative where applicable:

| **Inclusion** |  |  |
| --- | --- | --- |
| Age ≥ 65 years (≤49 for controls) | YES | NO |
| HIV-1 infected | YES | NO |
| Antiretroviral therapy with current ART ≥6months | YES | NO |
| **Visit 1** |  |  |
| Day of visit (yymmdd) |  |  |
| Current ART | Atazanavir  Darunavir  Efavirenz | Dose (mg/dose and number of doses/day): |
| Current backbone |  | Dose (mg/dose and number of doses/day): |
| Other current ART drugs |  | Dose (mg/dose and number of doses/day): |
| Time of sampling (yymmdd – hh:mm) |  |  |
| Time of last intake of ART (yymmdd-hh:mm) |  |  |
| Plasma sample taken | YES | NO |
| Serum sample taken | YES | NO |
| Adherence questionnaire completed | YES | NO |
| Adverse events/side effects form completed | YES | NO |
| Medication form completed (current/on-going medications only) | YES | NO |
|  |  |  |
| Year of birth (YYYY) |  |  |
| Sex | Female | Male |
| Concurrent Medical conditions | YES | NO |
|  | Cardiovascular disease  Cerebrovascular disease  Other neurological disease  Chronic kidney disease  Malignancy  Inflammatory disease  Diabetes  Hypertension  Immunosuppression  Other  AIDS defining diagnoses since first positive HIV test | Specify: |
| HIV-1 RNA (copies/ml) at day of visit |  |  |
| CD4 cell count (x10e6/L) at day of visit |  |  |
| CD4 cell count nadir (x10e6/L) |  | Date of sampling (yymmdd) |
|  |  |  |
| Chronic Hepatitis B | YES | NO |
| Chronic Hepatitis C | YES | NO |
| ALAT (μkat/L) |  |  |
| Bilirubin (μmol/L) |  |  |
| Creatinine (μmol/L) |  |  |
| Triglycerides (mmol/L) |  |  |
| LDL cholesterol (mmol/L) |  |  |
| Cholesterol (mmol/L) |  |  |
| Hemoglobin (g/L) |  |  |
| P-glucose (mmol/L) |  |  |
| Height (cm) |  |  |
| Weight (kg) |  |  |
|  |  |  |

| **Medication Form**  Generic name of drug (NB! ART drugs are reported elsewhere) | | Dose (mg/dose and number of doses/day) | | Date of initiation (yymmdd) | |
| --- | --- | --- | --- | --- | --- |
|  | |  | |  | |
|  | |  | |  | |
|  | |  | |  | |
|  | |  | |  | |
|  | |  | |  | |
|  | |  | |  | |
|  | |  | |  | |
| **Adverse event/ side effect form** |  | |  | |  |
| Symptom reported | Grade | | Date of onset (yymmdd) | | Relation to ART |
|  |  | |  | |  |
|  |  | |  | |  |

**Adherence questionnaire**

When was the last time you missed any of your medications?

Within the past week

1-2 weeks ago

2-4 weeks ago

1-3 months ago

More than 3 months ago

Never miss any medication

During the past four days, on how many days have you missed taking all your medication?

None

Two days

Three days

Four days
